# Transposition of the great arteries

**DOI:** 10.1186/1750-1172-3-27

**Published:** 2008-10-13

**Authors:** Paula Martins, Eduardo Castela

**Affiliations:** 1 Serviço de Cardiologia Pediátrica, Hospital Pediátrico de Coimbra, Coimbra, Portugal

## Abstract

Transposition of the great arteries (TGA), also referred to as complete transposition, is a congenital cardiac malformation characterised by atrioventricular concordance and ventriculoarterial (VA) discordance. The incidence is estimated at 1 in 3,500–5,000 live births, with a male-to-female ratio 1.5 to 3.2:1. In 50% of cases, the VA discordance is an isolated finding. In 10% of cases, TGA is associated with noncardiac malformations. The association with other cardiac malformations such as ventricular septal defect (VSD) and left ventricular outflow tract obstruction is frequent and dictates timing and clinical presentation, which consists of cyanosis with or without congestive heart failure. The onset and severity depend on anatomical and functional variants that influence the degree of mixing between the two circulations. If no obstructive lesions are present and there is a large VSD, cyanosis may go undetected and only be perceived during episodes of crying or agitation. In these cases, signs of congestive heart failure prevail. The exact aetiology remains unknown. Some associated risk factors (gestational diabetes mellitus, maternal exposure to rodenticides and herbicides, maternal use of antiepileptic drugs) have been postulated. Mutations in growth differentiation factor-1 gene, the thyroid hormone receptor-associated protein-2 gene and the gene encoding the cryptic protein have been shown implicated in discordant VA connections, but they explain only a small minority of TGA cases.

The diagnosis is confirmed by echocardiography, which also provides the morphological details required for future surgical management. Prenatal diagnosis by foetal echocardiography is possible and desirable, as it may improve the early neonatal management and reduce morbidity and mortality. Differential diagnosis includes other causes of central neonatal cyanosis. Palliative treatment with prostaglandin E1 and balloon atrial septostomy are usually required soon after birth. Surgical correction is performed at a later stage. Usually, the Jatene arterial switch operation is the procedure of choice. Whenever this operation is not feasible, adequate alternative surgical approach should be implemented. With the advent of newer and improved surgical techniques and post operative intensive care, the long-term survival is approximately 90% at 15 years of age. However, the exercise performance, cognitive function and quality of life may be impaired.

## Disease name and synonyms

Transposition of the great arteries; physiologically uncorrected transposition; complete transposition; atrioventricular concordance with ventriculoarterial discordance. The European paediatric cardiac code for this disease is 01.05.01.

## Definition and diagnostic criteria

The transposition of the great arteries was first described by Mathew Baillie in 1797, in the second edition of the book "*The Morbid Anatomy of Some of the Most Important Parts of the Human Body"*. However, the term transposition was only applied in 1814, by Farre, meaning that aorta and pulmonary trunk were placed (*positio*) across (*trans*) the ventricular septum.

In fact, this congenital cardiac malformation is characterised by atrioventricular concordance and ventriculoarterial discordance. In other words, the morphological right atrium is connected to the morphological right ventricle which gives rise entirely to or most of the aorta; the morphological left atrium is connected to the morphological left ventricle from where the pulmonary trunk emerges [1].

The term **congenitally corrected transposition **of the great arteries describes a different entity that conjugates atrioventricular and ventriculoarterial discordance [2]. In the old nomenclature, **completed transposition **was used to describe either congenitally corrected or uncorrected transposition. In both situations, the great arteries were completely or predominantly misplaced across the ventricular septum. The designation "incomplete transposition" was reserved to other types of malpositions of the great arteries, such as double outlet right and left ventricle. At present, the term complete transposition is usually used to describe only the physiologically uncorrected transposition.

The prefixes **a-, d- and l- transposition **describe the spatial relationship between the aorta and the pulmonary trunk, and should not be used to define this anomaly [3]. In d-transposition, the aortic valve lies to the right of the pulmonary valve. This is the most frequent arterial arrangement present in the hearts with concordant atrioventricular and discordant ventriculoarterial connections. However, other possibilities of arterial distribution exist in this setting, thus the two concepts are not synonyms [4]. In congenitally corrected hearts, the aorta usually lies on the left (l-transposition), but then again this is not an absolute finding. The a-transposition refers to the anterior position of the aortic valve in relation to the pulmonary trunk.

These different classifications and nomenclatures have lead in some cases to the inadequate or imprecise use of certain terms. To avoid ambiguity, it is important to use a description based on segmental analysis, and therefore, in our opinion, transposition of the great arteries should be defined as concordant atrioventricular and discordant ventriculoarterial connections.

## Epidemiology

The hearts with atrioventricular concordance and ventriculoarterial discordance represent 5–7% of all congenital heart diseases [5], corresponding to an incidence of 20 to 30 per 100 000 live births. There is a male predominance with a male/female sex ratio that varies, in the literature, from 1.5:1 to 3.2:1 [6-8]. In 10% of the cases, this cardiac lesion is associated with other noncardiac malformations [9].

## Pathology

In the hearts with transposition of the great arteries, atriums and ventricles retain their typical configuration, and conduction tissue assumes a similar distribution. A right-sided subaortic infundibulum usually separates the tricuspid from the aortic valve, and fibrous continuity is present between the mitral and the pulmonary valve. Coronary arteries' anatomy may assume diverse patterns of epicardial distribution, but they invariably originate in the aortic sinuses facing the pulmonary trunk. This fact is constant and independent of the two great vessels' spatial relation.

In 50% of the cases, the ventriculoarterial discordance is an isolated finding. This condition is designated as simple transposition. By contrast, complex transposition includes all the cases with coexisting malformations, such as ventricular septal defects, left ventricular outflow tract obstruction, aortic arch anomalies, and anomalous venous systemic return.

Ventricular septal defects are particularly common, and their location and size are variable. They may be associated with a certain degree of malalignement between the outlet and trabecular septum. If an anterior and rightward deviation of the outlet septum is present, concomitant pulmonary overriding and subaortic stenosis can be expected. In these cases, hypoplasia, coarctation, or even interruption of the aortic arch may be encountered. On the other hand, if the outlet septum is deviated posteriorly and leftwards, it may lead to subpulmonary stenosis [1].

In fact, left ventricular outflow tract obstruction is present in one-eighth to one-third of cases, being far more common in the presence of a ventricular septal defect than when the ventricular septum is intact. This impediment to the flow of blood can be caused by a wide spectrum of lesions, either at valvar or subvalvar level, and deviation of the muscular outlet septum is but one of them. Bulging of the septum, a fibrous shelf or fibromuscular tunnel, tissue tags, and anomalous attachment of the atrioventricular valvar tension apparatus can also be substrates for stenosis [10].

A careful assessment of all these morphological variants is of the uttermost significance, particularly for surgical planning and in foreseeing eventual complications.

## Pathophysiology

In the hearts with concordant atrioventricular and discordant ventriculoarterial connections, the systemic and pulmonary circulations run in parallel, rather than in series. As such oxygenated blood flows through a closed circuit that involves the lungs and the left cardiac chambers. On the other hand, systemic blood flow is supplied by another closed circuit that begins and ends in the right cardiac chambers. In this setting, survival is only possible, if there is adequate mixing between the two circulations, be it between the septums or through the arterial duct[11].

## Clinical manifestations

The parallel circulation just described results in a significant hypoxemic status that is observed clinically by central cyanosis. The bluish discoloration of the skin and mucous membranes is therefore the basic pattern of clinical presentation in transposition. Its onset and severity depend on anatomical and functional variants that influence the degree of mixing between the two circulations.

Limited intercirculatory mixing, usually present if the ventricular septum is intact or the atrial septal defect is restrictive, is related to progressive and profound central cyanosis evident within the first hours of life. Likewise, associated left ventricular outflow tract obstruction or pulmonary obstructive disease can reduce the blood flow to the pulmonary vascular bed, and thus contributing to a marked cyanotic state. However, if no obstructive lesions are present, and there is a large ventricular septal defect that allows for satisfactory mixing between the two circulations, cyanosis may go undetected and only be perceived during episodes of crying or agitation. In these cases, signs of congestive heart failure prevail due to excessive ventricular workload. Tachypnoea, tachycardia, diaphoresis, poor weight gain, a gallop rhythm, and eventually hepatomegaly can be then detected later on during infancy.

Heart murmurs associated with left outflow tract obstruction, persistent arterial duct or due to a septal defect may be heard, but they are not a constant finding.

## Aetiology

The exact aetiology of this disease is still unknown. However, some associated risk factors, namely gestational diabetes mellitus [12,13], maternal exposure to rodenticides and herbicides [14], and maternal use of antiepileptic drugs [15] have been postulated.

Significant advances in the understanding of the underlying genetic mechanisms have been achieved over the last decade. Several mutations have been implicated as the cause of discordant ventriculoarterial connections. The genes involved so far are the growth differentiation factor-1 gene [16], the thyroid hormone receptor-associated protein-2 gene [17], and the gene encoding the cryptic protein [18]. They are localised in different chromosomes and their mutations only explain a small minority of the clinical cases.

## Diagnostic methods

Diagnosis involves careful history taking and physical examination of the patient under good light condition. When these and the **hyperoxia test **are suggestive of a cyanotic congenital heart condition, further investigations are required.

A **chest X-ray **and an **electrocardiogram **may be helpful at this stage, but their respective findings are not specific. In transposition, a narrowed superior mediastinum gives to the cardiac silhouette a characteristic egg-shaped appearance on chest radiography. Cardiomegaly with increased pulmonary vascular markings may be found if a ventricular septal defect is present. The main electrocardiographic features are a rightward deviation of the QRS complex axis associated with right ventricular hypertrophy; biventricular hypertrophy is evident in conditions that lead to a left ventricular overload.

**Figure 1:**
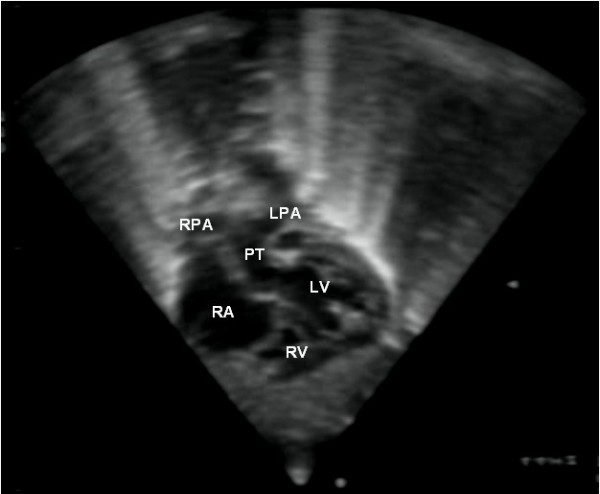
This subcostal view shows the left ventricle originating a vessel that bifurcates, which is thus identified as the pulmonary artery.

**Figure 2:**
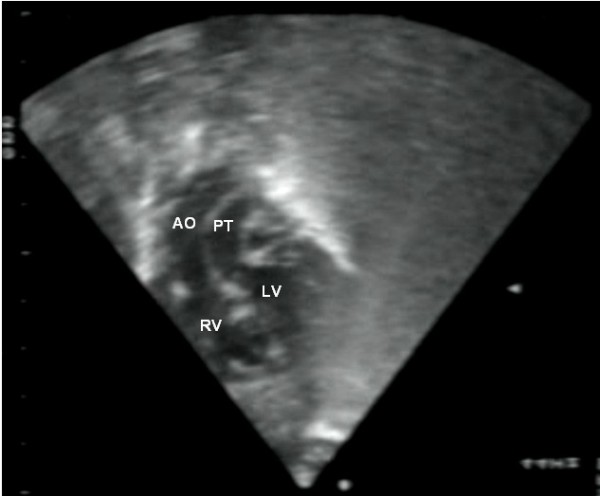
Subcostal view showing discordant ventriculoarterial connections together with the presence of parallel, rather than crossing, great arteries arising form the ventricles.

**Figure 3:**
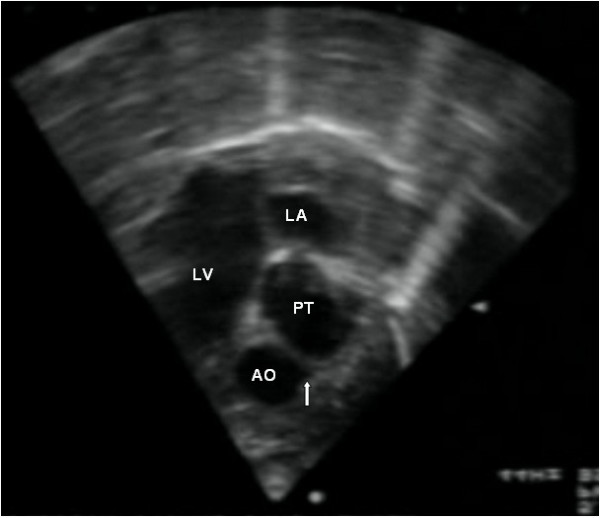
**Short axis view showing the aorta giving rise to the coronary arteries (arrow) in an anterior position and to the right.** The pulmonary trunk is placed in a central position.

The definitive diagnosis relies however on **echocardiography**. This imaging modality provides accurate morphological and functional assessment of the heart, being able to show the specific features of the transposition of the great arteries. In the four-chamber view, one assesses atrioventricular concordance, but the ventriculoarterial discordance is better observed using other incidences. In the five-chamber, parasternal long-axis or even subcostal view, the vessel arising from the morphologically left ventricle has a posterior course and bifurcates immediately, being identified as the pulmonary trunk (Fig. 1). The morphologically right ventricle is related to a vessel that gives out the coronary, head and neck arteries, thus the aorta. The proximal portions of the two arteries run parallel to each other, rather than in the usual cross pattern, giving it a typical and easily recognisable appearance in the parasternal long-axis and subcostal views (Fig. 2). In the short-axis view, the pulmonary trunk is usually in a central position, with the aorta being placed anteriorly and to the right (Fig. 3).

**Figure 4:**
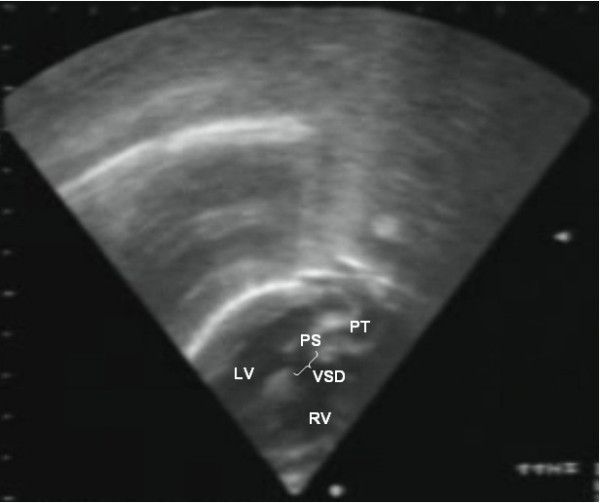
Echocardiography of a complex transposition with a ventricular septal defect and pulmonary stenosis.

The evaluation of the coronary artery pattern [19,20] and exclusion of other malformations are two important aspects that need to be addressed prior to surgery (Fig. 4). Furthermore, the Doppler study also provides functional information and complements the data provided by the two-dimensional echo. Flow through the arterial duct and septal defects are visualised, being an indicator of the adequacy of mixing between the systemic and pulmonary circulations. Moreover, it is possible to measure gradients through obstructive lesions and to assess the function of the atrioventricular and semilunar valves.

As echocardiography in experienced hands is a reliable diagnostic tool providing high sensivity and specificity, the need for **catheterisation **is limited to cases that require clarification of certain anatomic and haemodynamic aspects not clearly identified by echocardiography [21-24]. Likewise, **CT **or **MR imaging **can also offer additional details of some associated lesions.

**Prenatal diagnosis **by foetal echocardiography is possible and desirable, as it may improve early neonatal management, thus reducing related morbidity and mortality [25,26]. This method has proven to be very accurate, not only in the diagnosis of the disease and associated malformations, but also in planning the most appropriate surgical approach [27].

## Differential diagnosis

Differential diagnosis should include other causes of central neonatal cyanosis. In this clinical setting, the hyperoxia test provides a simple means of assessing preductal PaO2 response to 100% inspired oxygen, aiding in the differentiation between cyanosis caused by cardiac disease from that caused by neurological or pulmonary disorders.

In the neonates with parallel circulation, on a 100% inspired oxygen, a blood sample collected from the right radial artery will typically show a PaO2 less than 50 mmHg. In this case, further exams should be carried out in order to confirm the diagnosis.

## Management

### Palliative treatment

The initial aim in the management of the affected newborns is to assure acceptable intercirculatory mixing. The presence of an atrial or a ventricular septal defect that provides satisfactory mixing, permits corrective surgery at a later stage without the prior need for palliative procedures. Nevertheless, this is not the most common situation, and usually a first stage treatment is required.

Intravenous **prostaglandin E1 **infusion is used to maintain arterial duct patency leading to an increment in pulmonary blood flow, which increases pulmonary venous return and left atrial pressure, thus promoting left to right flow at atrial level [28]. Early and late side effects may be observed, namely apnoeas, bradycardia, systemic hypotension, fluid-electrolyte disturbances, fever and cutaneous flushing. Long-term use is associated with cortical hyperostosis, an effect that does not seem to be dose-related. Monitoring in an intensive care unit is therefore advised [29].

Although intercirculatory mixing improves, prostaglandin action is frequently modest and insufficient to assure a satisfactory oxygenation of the systemic blood, either in simple or complex transposition. **Balloon atrial septostomy**, also known as the Rashkind procedure, assumes therefore an important role in the pre-operative management of these babies. This technique involves the placing of a balloon-tipped catheter in the left atrium, *via *the oval foramen. The balloon is then inflated and pulled back into the right atrium, tearing the atrial septum. In some centres, this procedure is echocardiographically guided providing reliable visual guidance without exposure to ionising radiation. Thus, the risk of complications is minimised and the final diameter of the interatrial orifice can be accessed [30]. The result is considered successful when an atrial septal defect with at least 5 mm in diameter, an increased flapping motion of the inferior rim of the atrial septum is observed and there is an increase in the oxygen saturation [31].

Balloon septostomy is an effective and safe procedure for creating long lasting adequate interatrial communications [32]. It has largely replaced other more aggressive interventions such as blade atrial septostomy and surgical atrial septectomy. At the present, their use is limited to specific situations, mainly related to the rigidity of the interatrial septum. Balloon dilatation may also be required, especially in older patients in whom good palliation was not achieved by usual septostomy.

In addition to these measures, medical support is usually necessary to optimise the clinical condition. Mechanical ventilation and oxygen may be needed in the unstable newborn with severe hypoxaemia. Nevertheless, it should be emphasised that these measures are not universally beneficial to these patients. For example, oxygen therapy may promote ductal closure, compromising intercirculatory mixing. Futhermore, aggressive ventilator settings (Positive Inspiratory Pressure and Positive End Expiratory Pressure) leading to excessive alveolar distension should be avoided so that intercirculatory shunts are not disturbed.

The correction of metabolic acidosis with bicarbonate is important, as it may compromise myocardial function and in case of cardiac failure, inotropic agents or diuretics may be necessary.

### Corrective treatment

The **arterial switch **operation is the procedure of choice used to achieve complete physiological and anatomical repair. Its supremacy has been corroborated by long-term results that show preservation of good left ventricular function, sinus rhythm and a low mortality with a survival rate of 88% at both 10 and 15 years. The rare post operative complications are mainly related to prolonged peri-operative ischaemia times, aortic regurgitation and coronary artery obstruction, which may result in myocardial ischaemia or even infarction. A low reoperation rate has also been reported, pulmonary stenosis at the site of reconstruction being the most common cause [33].

**Figure 5:**
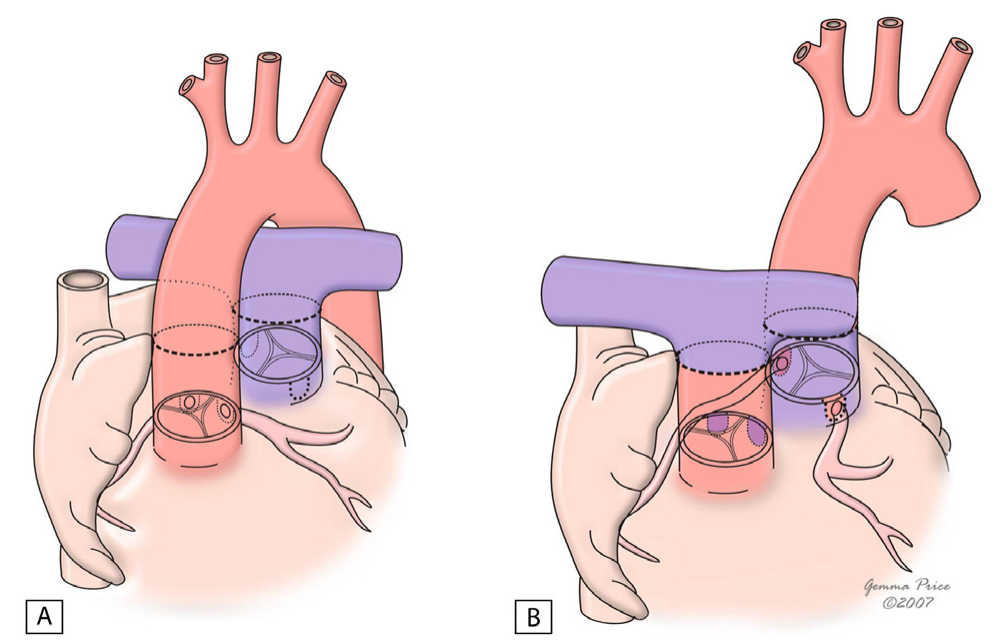
**The figure illustrates the arterial switch procedure.** It shows the connection of the proximal great arteries to the distal end of the other great artery, as well as the transfer of coronary arteries to the new aorta. (adapted from [10]. Martins P, Tran V, Price G, Tsang V and Cook A, Cardiol Young 2008; 18:124–34, with permission from Cambridge University Press).

In this surgery, the aorta and pulmonary trunks are sectioned and their distal extremities are transposed and anastomosed; coronary arteries are then translocated to the neo-aorta (Fig. 5). The correction should be ideally performed in the first month of life. Several factors may however interfere with this timing, namely missed diagnosis and preoperative complications, such as multiorgan failure, renal failure, active infection, severe acidosis or subarachnoidian haemorrahage, that postpone the procedure until the clinical condition is more stable. On the other hand, if palliative measures have had poor results or resulted in untoward complications, an emergency arterial switch maybe needed sooner.

In the specific context of transposition with intact septum, the newborn should be operated preferably within the first two-weeks of life. This would ensure that left ventricle has not suffered significant involution, and its contractility is still able to support the systemic circulation. In older neonates and young infants, the left ventricle may need to be retrained, before the arterial switch is attempted, by pulmonary artery banding, with or without an associated Blalock-Taussig shunt. This intermediate step should establish a left/right ventricular pressure ratio between 0.6/0.75 without compromising the required pulmonary blood flow [34]. This pressure ratio is a known inducer of left ventricle remodelling. The exact indications for the two-stage arterial switch are still controversial, and, besides patient age, other factors should be considered, such as left ventricular wall thickness, left ventricular volume and corresponding estimated mass, left ventricular pressure and the degree of interventricular septum bounding [34-36]. According to existing evidence, a short time interval between the two operations [37], usually one week, is sufficient. Nevertheless, the decision to proceed to the arterial switch is based on the evolution of the echocardiografic parameters concerning both left ventricle structure and haemodynamic performance. The results reported appear to be very encouraging, showing a survival rate and freedom of reoperation at five years of 90% and 97%, respectively [38].

In complex transposition, the arterial switch procedure should be tailored towards the individual morphological aspects and complementary interventions may be necessary to repair concomitant malformations. In fact, an associated atrial defect, or more commonly the septostomy defect, can usually be closed by direct suture. Closure of the ventricular septal defect may require the use of a patch to close the communication or, if very small, may just be left open. An obstruction within the aortic arch is best repaired concomitantly, if necessary using a pulmonary homograft to enlarge the aorta [39]. Some lesions causing left outflow tract obstruction may be effectively ressected, re-establishing an adequate patency. Complex coronary patterns may require individualized techniques for coronary transfer, adapted to the ostial anatomy and coronary course.

Two morphologic features may preclude however the use of this operation: an outflow tract obstruction lesion that can not be satisfactory relieved by resection and certain coronary artery patterns that may pose difficulties during coronary transfer.

**Figure 6:**
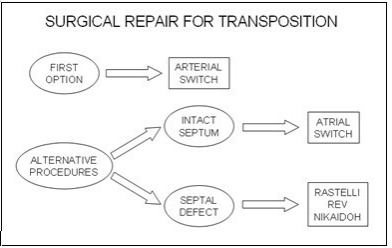
Surgical procedures used in the correction of the transposition of the great arteries.

**Figure 7:**
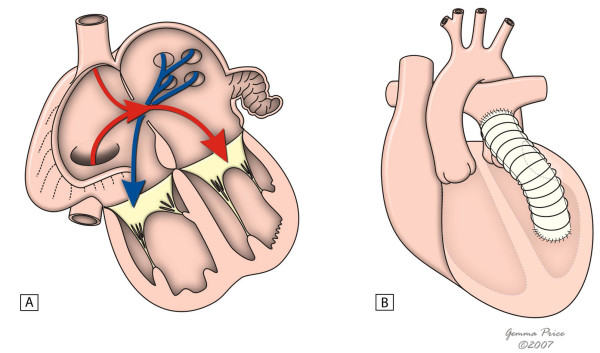
**This figure shows the common principle subjacent to intra-atrial switch.** Figure 7A shows in diagrammatic fashion, the redirection of pulmonary and systemic venous flows at atrial level. An external conduit (B) is placed to overcome the obstruction within the left ventricular outflow tract. (adapted from [10]. Martins P, Tran V, Price G, Tsang V and Cook A, Cardiol Young 2008; 18:124–34, with permission from Cambridge University Press).

Whenever the arterial switch is not feasible, alternative approaches are required – Fig. 6[10]. A repair at atrial level, either by a Mustard or a Senning procedure, is particularly suitable for hearts with an intact ventricular septum. The systemic venous return is redirected at atrial level to the left ventricle. Likewise, pulmonary venous blood is diverted to the right ventricle. When there is an obstruction at the left ventricular outflow tract, an extracardiac conduit is placed between the left ventricle and the pulmonary trunk- Fig. 7[40]. The main complications of these techniques are sinus node dysfunction, obstruction to either pulmonary or systemic venous return, supraventricular tachyarrythmias, residual interatrial shunt, right ventricular dysfunction and pulmonary vascular obstructive disease. These factors are associated with a less satisfactory survival rate of 77.7% and 67.2% at 10 and 30 years respectively, with an early mortality accounting for 16% [41]. Particularly adverse outcomes are present in patients with an advanced New York Heart Association functional class or with arrythmias [42].

**Figure 8:**
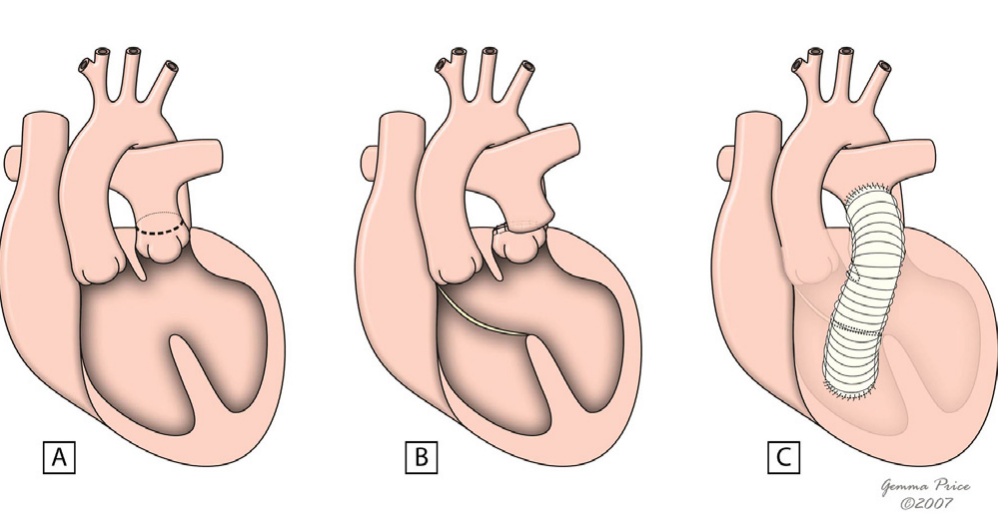
**The figure shows the main steps of Rastelli procedure in the setting of a deviated outlet septum (A).** A patch is placed to create an interventricular tunnel (B), and an extracardiac conduit is placed between the right ventricle and the pulmonary arteries (C). (adapted from [10]. Martins P, Tran V, Price G, Tsang V and Cook A, Cardiol Young 2008; 18:124–34, with permission from Cambridge University Press).

**Figure 9:**
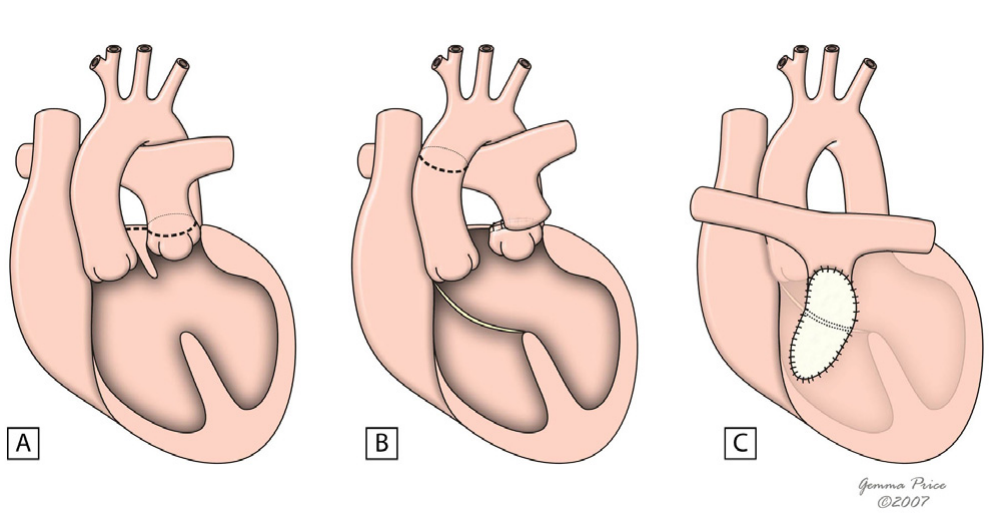
**This figure shows the innovations of the REV procedure relative to the Rastelli procedure, with resection of the muscular outlet septum (B) and use of Lecompte manoeuvre (C) which avoids the use of an extacardiac conduit.** (adapted from [10]. Martins P, Tran V, Price G, Tsang V and Cook A, Cardiol Young 2008; 18:124–34, with permission from Cambridge University Press).

In the presence of a ventricular septal defect, the most used options are the REV procedure or its modification and the Rastelli operation. In both, an intraventricular tunnel passing through the septal defect is created to connect the left ventricle to the aorta. In the Rastelli procedure, an extracardiac conduit is placed connecting the right ventricle to the pulmonary artery (Fig. 8). However, in the REV technique, this is accomplished through the LeCompte manouever, which brings the pulmonary trunk forward, allowing its direct implantation in the right ventricle (Fig. 9). Apart from avoiding the use of an extracardiac conduit, the REV procedure has a further advantage in which it involves the resection of the muscular outlet septum, providing better alignment between the aorta and the left ventricle. As expected, late results in terms of reoperation are significantly different in the two procedures. In fact, Rastelli operation was associated with a greater risk of reintervention due to left ventricular outflow tract obstruction, and extracardiac conduit problems such as obstruction, requiring eventual replacement. However, similar early and late mortalities were reported [43,44].

**Figure 10:**
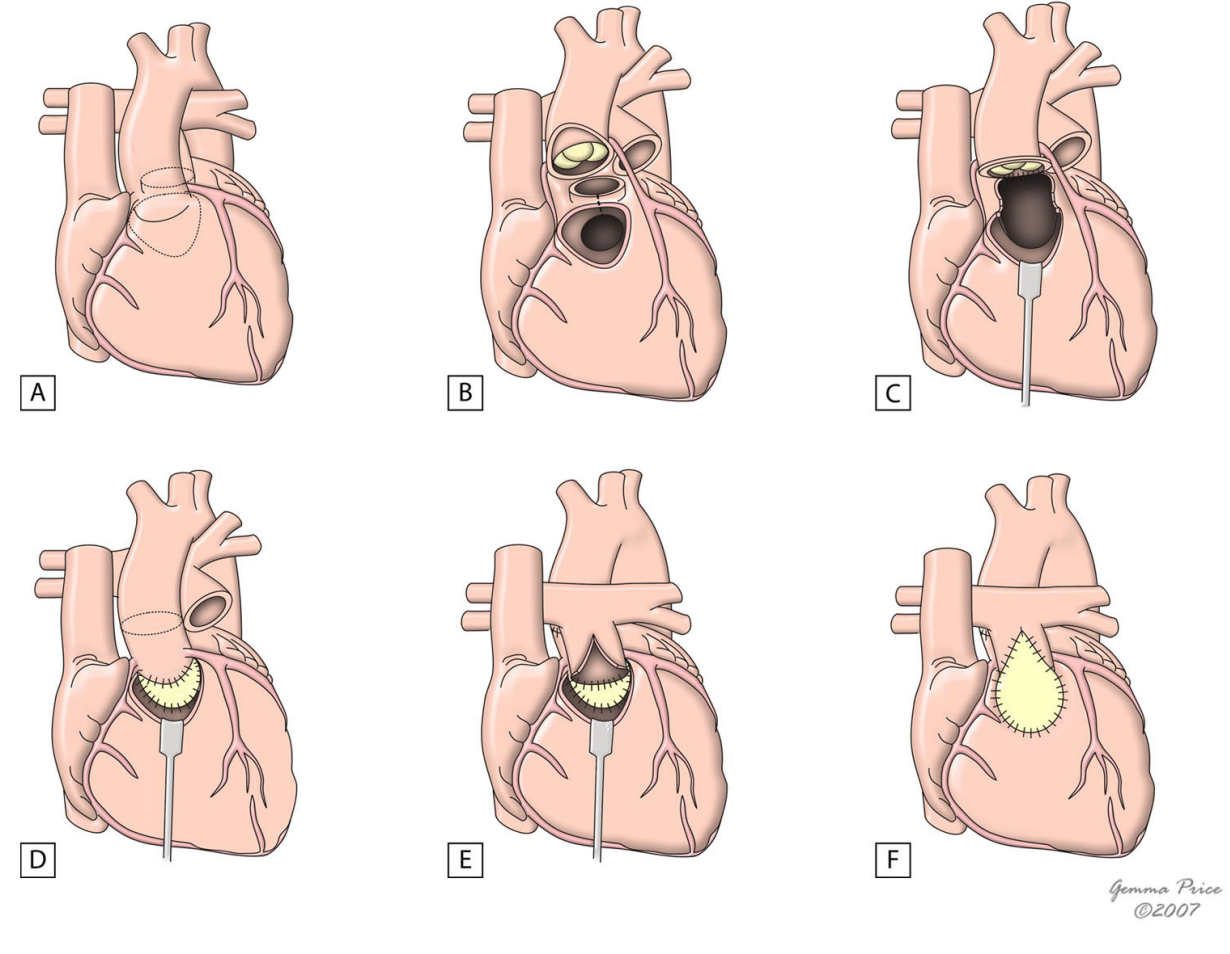
**The figure shows the major issues of Nikaidoh's procedure.** Figure 10A shows the areas for harvesting the aortic root and transection of the pulmonary trunk. In 10B, the aorta and pulmonary trunk have already been disconnected from their previous insertion, and the line of division of the outlet septum is shown. Figures 10C and 10D show aortic translocation and reconstruction of the left outflow tract, with a pericardial patch used to close the ventricular septal defect. In 10E and 10F, the pulmonary trunk is sutured to the right ventricular outflow tract, and the reconstruction is completed with a pericardial patch. (adapted from [10]. Martins P, Tran V, Price G, Tsang V and Cook A, Cardiol Young 2008; 18:124–34, with permission from Cambridge University Press).

The Nikaidoh's procedure is also an option in the setting of discordant ventriculoarterial connections, ventricular septal defect and left outflow tract obstruction, especially if inadequate anatomy for a REV or Rastelli is present [45]. The repair is achieved by removing the aortic root with its coronaries attached, and translocating them to the prior pulmonary position. The obstruction in the left ventricular outflow tract is relieved through outlet septum bisection and resection of the pulmonary valve. Both ventricular outflow tracts are then reconstructed, using patches either to close the ventricular septal defect or to adapt pulmonary trunk to the right infundibular area (Fig. 10). The major handicaps of this approach are its technical difficulty, as well as a relatively high rate of reoperation due to right ventricular outflow tract obstruction and pulmonary insufficiency; moreover, unusual coronary patterns may also represent an additional problem. Despite all these disadvantages, a recent study comparing REV, Rastelli and Nikaidoh's performances has highlighted the superiority of Nikhaidoh's approach in obtaining a better physiologic cardiac haemodynamics [46] (Table 1) [47-49]. Further studies are however necessary to perfectly establish the role of this surgical approach in transposition correction.

**Table 1:** Different types of corrective surgeries and respective early mortality, late survival, complications and probability of reoperation

Type of surgery	Era	Early mortality	Late survival	Major complications and incidence	Reoperations
*First choice*					
• Arterial switch [33,47]	1975	3.8%	88% (10 and 15 yrs)	Pulmonary stenosis (3.9%) Aortic regurgitation (3,8%) Coronary lesions (2%)	4.5–18%

*Alternative procedures*					
• Simple TGA					
- Atrial switch [41,42]	1960s	16.5%	77.7% (10 yrs) 67.2% (30 yrs)	Arrythmia (47.6 – 64.3%) Tricuspid regurgitation (34.9%) Systemic ventricular dysfunction (11.5–14.6%) Baffle related problems (5.6%)	5.1%

• TGA with VSD ± LVOTO					
- Rastelli [48]	1969	7%	80% (10 yrs) 52% (20 yrs)	RVOTO (64%) Arrythmias (24%) LVOTO (16%)	44%
- REV [43]	1982	12.5%	84% (5 yrs)	RVOTO (19%)	10%
- Nikaidoh [49]	1984	5%	95%	RVOTO (26%)	47%

### Prognosis

Until mid twentieth century, the treatment of transposition was restricted to few palliative measures and the natural history of the disease with its poor prognosis was an undeniable reality. By that time, the average life expectancy for a newborn with transposition was 0.65 years and the mortality rate at one year was 89.3% [50]. With the advent of newer and improved surgical techniques as well as post operative intensive care, the scenario has completely changed, and very encouraging long-term survival rates almost achieving 90% at 15 years of age have been reported. The potentialities of the current corrective surgery modalities are also underlined by a low 10-year re-intervention rate (6%) and a corresponding event-free survival of 88% [51]. Nevertheless, recent studies have pointed out to a reduced exercise performance, a compromise in cognitive functioning, and an unfavourable health-related quality of life [51,52]. Further improvements are therefore necessary and they may be achieved in the future by reinforcing prenatal diagnosis and by establishing strategies to minimise surgical complications.

## Competing interests

The authors declare that they have no competing interests.

## Authors' contributions

The authors equally contributed to this review article. They read and approved the final version of the manuscript.
